# Notch Signaling Activation Suppresses v-Src-Induced Transformation of Neural Cells by Restoring TGF-β-Mediated Differentiation

**DOI:** 10.1371/journal.pone.0013572

**Published:** 2010-10-22

**Authors:** Samira Amarir, Maria Marx, Georges Calothy

**Affiliations:** CNRS UMR3347/INSERM U1021, Institut Curie-Section Recherche, Orsay, France; Duke University, United States of America

## Abstract

**Background:**

We have been investigating how interruption of differentiation contributes to the oncogenic process and the possibility to reverse the transformed phenotype by restoring differentiation. In a previous report, we correlated the capacity of intracellular Notch (ICN) to suppress v-Src-mediated transformation of quail neuroretina (QNR/v-src^ts^) cells with the acquisition by these undifferentiated cells of glial differentiation markers.

**Methodology/Principal Findings:**

In this work, we have identified autocrine TGF-β3 signaling activation as a major effector of Notch-induced phenotypic changes, sufficient to induce transition in differentiation markers expression, suppress morphological transformation and significantly inhibit anchorage-independent growth. We also show that this signaling is constitutive of and contributes to *ex-vivo* autonomous QNR cell differentiation and that its down-regulation is essential to achieve v-Src-induced transformation.

**Conclusions/Significance:**

These results support the possibility that Notch signaling induces differentiation and suppresses transformation by a novel mechanism, involving secreted proteins. They also underline the importance of extracellular signals in controlling the balance between normal and transformed phenotypes.

## Introduction

Differentiation is a multi-step process resulting from a cascade of alternate activation and extinction of tissue-specific signaling pathways. Progression through this cascade is generally mediated by a sequence of extracellular signals, initiated by the differentiating cells themselves or by their environment. As a consequence of oncogenic events, this process is often interrupted and differentiating cells can no longer exit the cell cycle. Hence, blocking differentiation constitutes an important step in neoplastic transformation. However, understanding its contribution can be achieved only through the use of adequate experimental models. The rationale for a differentiation therapy is based on the assumption that cancer cells have retained the potential to respond to appropriate differentiation signals, which in turn would be sufficient to restore a normal phenotype. This could be achieved either because the transformed cells undergo growth arrest or because they no longer respond to oncogenic stimuli. Developing models for the latter would be useful to identify pathways essential for transformation and possibly result in new therapeutic approaches. However, the task of reverting a cancer cell to its normal state, in response to differentiation signals, has been only reached in a very few clinical or experimental instances (for review, [1]).

We previously reported that stable expression of Notch intra cellular domain (ICN) suppresses transformation of embryonic quail neuroretina (QNR) cells induced by a temperature sensitive v-Src (QNR/v-src^ts^), without altering oncoprotein expression nor its downstream signaling activity. This remarkable phenotypic change is correlated with a differentiation switch, as these undifferentiated transformed cells acquire markers of glial cells [2]. Several reasons support the choice of this *ex-vivo* model to study how activation of differentiation signals could result in transformation suppression. QNR cells dissected from 7-day old embryos progressively cease to divide and autonomously execute glial and neuronal differentiation programs [2]–[4]. As a consequence of v-Src activity, they acquire sustained proliferative capacity [5], [6], display all characteristics of oncogenic transformation [7], [8] and repress their autonomous differentiation potential [2], [9], [10].

We have selected Notch as an instructive signal, because of its important contribution to neuroretina development. At early stages, it maintains progenitor cells in an undifferentiated state by inhibiting their neuronal differentiation [11], whereas at later stages it promotes glial differentiation [12], [13]. We were also interested in this signaling pathway because of its dual contribution to either oncogenesis or tumor suppression, depending on the cell model (for review, [14]). We also showed that both suppression of transformation and switch in differentiation markers expression were mediated by its transcription factor partner, CBF [2]. Therefore, these results demonstrated that activating differentiation signals was sufficient to abolish cell response to oncogenic stimuli, thus lending further experimental basis to the differentiation therapy concept.

Our previous work also indicated that interference of constitutive Notch signaling with transformation possibly involved a secreted factor(s). Culture medium from revertant cells, stably expressing the Notch intracellular domain (QNR/v-src^ts^/ICN) or an activated human CBF (RBPJ-k), contains a paracrine activity which inhibits transformation of QNR/v-src^ts^ cells [2]. This suggested that secreted factors could play a key role at the cross-roads between transformation and differentiation, in this cell system. Therefore, we undertook to identify this activity and investigate its possible autocrine effect on QNR cell transformation and differentiation.

In this report, we identified autocrine activation of TGF-β3 signaling as a major effector of the phenotypic changes induced by ICN signaling, sufficient to suppress transformation of QNR/v-src^ts^ cells and promote their acquisition of glial differentiation markers, in presence of an active oncoprotein. We also show that this signaling is activated during QNR cell *ex-vivo* differentiation and that its down-regulation by v-Src is essential to block differentiation and achieve transformation. Taken together, our results provide a potentially novel mechanism by which Notch signaling suppresses oncogenic transformation. They also underline the importance of extracellular signals in maintaining the balance between the normal and transformed phenotypes.

## Results

### TGF-β3 mRNA is upregulated in QNR/v-src^ts^ cells stably expressing ICN

To understand the mechanisms by which Notch signaling activation suppressed cell transformation, we compared the transcription profile of QNR cells transformed by a v-src mutant encoding a temperature sensitive (ts) oncoprotein (QNR/v-src^ts^), with that of cells stably expressing ICN (QNR/v-src^ts^/ICN). mRNA were prepared from cells maintained at permissive (37°C) or restrictive (41°C) temperature. For each temperature, cDNA from QNR/v-src^ts^ cells were probed with that of QNR/v-src^ts^/ICN cells, on microarrays spotted with 13,000 cDNA from chicken EST collections [15]. This analysis revealed 209 genes displaying, at least, a 1.5 fold increase in QNR/v-src^ts^/ICN, as compared to QNR/v-src^ts^ cells. As we previously showed that suppression of QNR/v-src^ts^ cell transformation possibly involved a secreted factor(s) [2], we were primarily interested in genes encoding potentially secreted proteins. Among 18 such genes, we identified, as a most highly induced one, the gene encoding the TGF-β3 ligand, the expression of which was increased 8 to 16 times in QNR/v-src^ts^/ICN cells. We focused our study on this protein, because of its important role during oncogenesis (for review, [16]).

**Figure 1:**
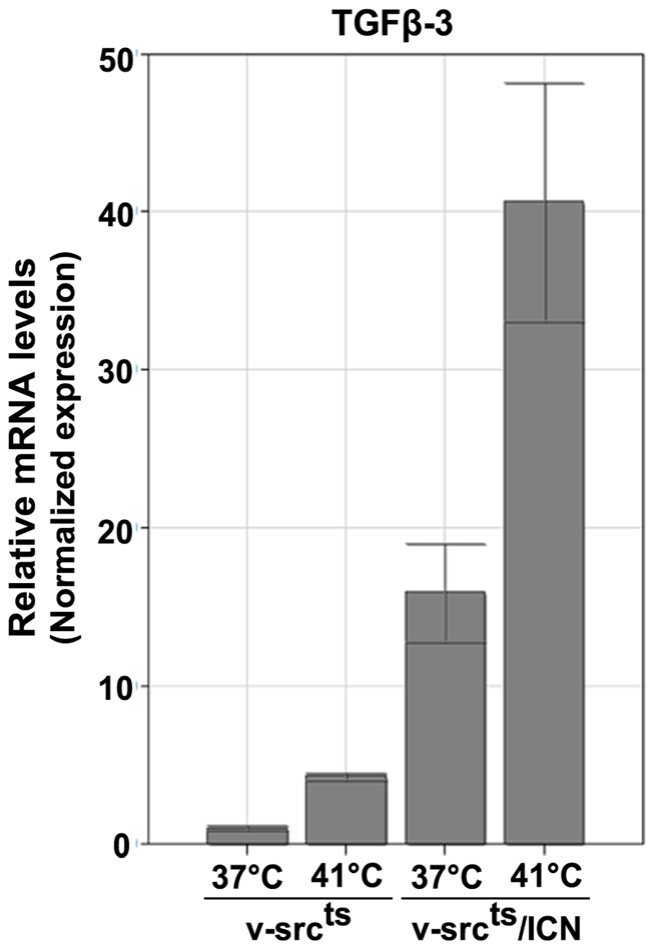
TGF-β3 mRNA is upregulated in QNR/v-src^ts^/ICN cells. QNR/v-src^ts^ and QNR/v-src^ts^/ICN cells were incubated 72 hrs at 37°C or 41°C before extraction of total RNA. After reverse transcription of 1 µg of RNA, TGF-β3 cDNA was amplified by QPCR using specific primers (Table 1) Results were normalized based on HPRT and TBP transcript levels and presented in relative arbitrary units. Normalized TGF-β3 mRNA levels detected in QNR/v-src^ts^ cells at 37°C were used as reference equal to 1. Each bar represents the mean -/+ S.E. of experiments realized on 3 different cultures per cell type.

Gene array results were validated by QPCR experiments. These experiments showed a 13-time induction of normalized TGF-β3 mRNA levels in QNR/v-src^ts^/ICN cells, compared to QNR/v-src^ts^ cells at 37°C. At 41°C, TGF-β3 mRNA amounts were 10 times higher in QNR/v-src^ts^/ICN cells than in QNR/v-src^ts^ cells, at the same temperature (Fig. 1). However, in QNR/v-src^ts^/ICN cells, transcript levels were more elevated at 41°C than at 37°C. A similar difference was observed in QNR/v-src^ts^ cells, suggesting that TGF-β3 mRNA levels were regulated by v-Src activity. Increased levels of TGF-β3 mRNA were also found in QNR/v-src^ts^ cells stably expressing a constitutively activated human RBP-Jk, indicating that this increase was mediated by the ICN/CBF complex (data not shown). Therefore, it was likely that the presence of higher TGF-β3 mRNA levels would lead to an activation of TGF-β signaling in QNR/v-src^ts^/ICN cells.

### QNR/v-src^ts^/ICN cells secrete and respond to mature TGF-β3 activity

**Figure 2:**
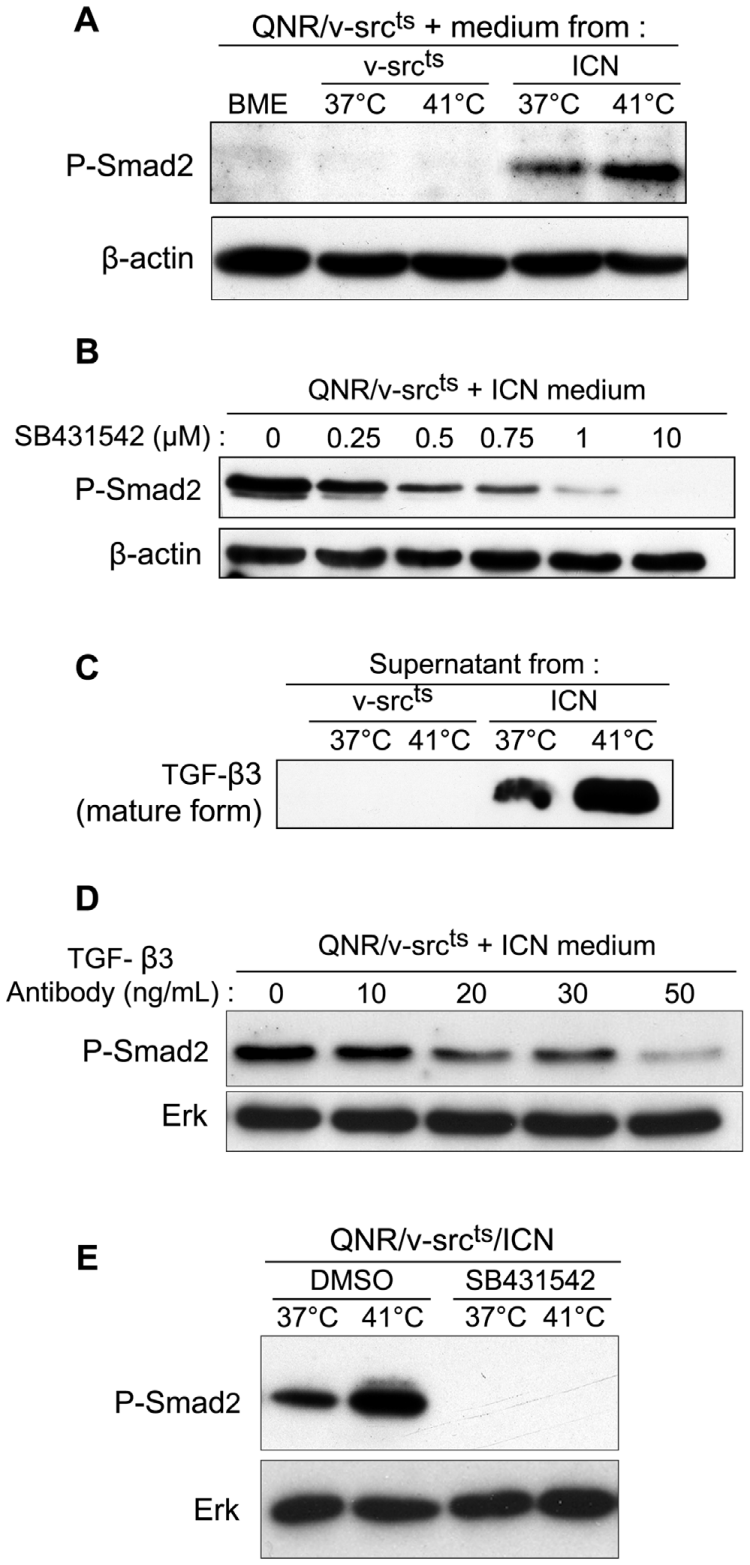
QNR/v-src^ts^/ICN cells secrete and respond to mature TGF-β3. (A) Media were collected after a 30-hour incubation of QNR/v-src^ts^ (v-src^ts^ medium) and QNR/v-src^ts^/ICN (ICN medium) cells at 37°C or 41°C. QNR/v-src^ts^ cells were incubated at 37°C in normal medium (BME) or conditioned media for 1 hour, before lysis. Phosphorylated Smad2 was detected by western-blot. β–actin was used for protein level normalization. (B) QNR/v-src^ts^ cells were treated at 37°C with increasing concentrations of SB431542 inhibitor for 2 hrs and then incubated in ICN medium during 1 hour in presence of inhibitor. Effects of this treatment on Smad2 phosphorylation were analyzed by western-blot. (C) Comparison of mature TGF-β3 levels in v-Src and ICN media. Cells were plated at 2.10^6^ cells per 100 mm dish at 37°C or 41°C. One day after plating, growth media were replaced with serum-free BME. After a 30-hour incubation, media were collected and concentrated 50 times using Vivaspin6 columns. 20 µg of proteins were migrated under non-denaturing conditions. Mature TGF-β3 migrates as 25 kDa band. (D) QNR/v-src^ts^ cells were treated, one hour before lysis, with ICN medium preincubated at 37°C for one hour with increasing amounts of neutralizing antibodies directed against TGF-β3. Levels of Smad2 phosphorylation were analyzed by western-blot. Erk was used to normalize protein loading. (E) Levels of phosphorylated Smad2 in QNR/v-src^ts^/ICN cells treated at 37°C or 41°C with DMSO or 10 µM of SB431542 inhibitor for 24 hrs.

We first investigated whether mature TGF-β was secreted by QNR/v-src^ts^/ICN cells. Therefore, we collected cell-free medium from these cells (ICN medium) maintained at 41°C (v-Src inactive) and from cells transferred to 37°C (v-Src active) for 72 hours, and examined its capacity to activate phosphorylation of the TGF-β signaling effector Smad2 [17], in QNR/v-src^ts^ cells. Control medium was obtained from transformed QNR/v-src^ts^ cells (v-Src medium), maintained at either temperature. Phosphorylated Smad2 was barely detectable in QNR/v-src^ts^ cells cultured at 37°C and was not increased when these cells were treated with their own medium collected at either temperature (Fig. 2A), ruling out both TGF-β signaling activation in transformed cells and TGF-β release by these cells at detectable levels. In contrast, incubating QNR/v-src^ts^ cells in ICN medium, collected at either temperature, markedly increased the levels of phosphorylated Smad2. Furthermore, in agreement with our comparative Q-PCR results, medium collected at 41°C was significantly more efficient in activating Smad2 phosphorylation (Fig. 2A).

To confirm that this increase in Smad2 phosphorylation was due to TGF-β activity, we treated QNR/v-src^ts^ cells with ICN medium in presence of the TGF-βRI kinase inhibitor, SB431542 [18], at concentrations ranging between 0.25 and 10 µM. We observed a dose dependent reduction of Smad2 phosphorylation in presence of the inhibitor (Fig. 2B).

In agreement with transcriptome analysis, we detected, by Western blotting, the presence of a specific mature TGF-β3 band, migrating at about 25 kDa, in ICN medium, collected at either temperature (Fig. 2C). This band was present in higher amounts in ICN medium collected at 41°C and was not found in v-Src medium collected under either temperature condition. To confirm the contribution of TGF-β3 to Smad2 phosphorylation, we incubated QNR/v-src^ts^ cells with ICN medium, in the presence of increasing amounts of TGF-β3 blocking antibody. In agreement with SB431542 treatment, the levels of phosphorylated Smad2 were decreased as a function of antibody concentration (Fig. 2D), indicating that Smad2 phosphorylation was essentially due to secreted TGF-β3.

Finally, we examined the activation of TGF-β signaling in QNR/v-src^ts^/ICN cells by measuring endogenous Smad2 phosphorylation in presence or absence of an active v-Src. Phosphorylated Smad2 was detected in QNR/v-src^ts^/ICN cells at 37°C and at higher levels at 41°C (Fig. 2E). In both cases, the presence of phosphorylated Smad2 was sensitive to that of the SB431542 inhibitor (Fig. 2E), indicating that activation of TGF-β signaling resulted from an autocrine response of these cells to mature TGF-β3 activity.

**Figure 3:**
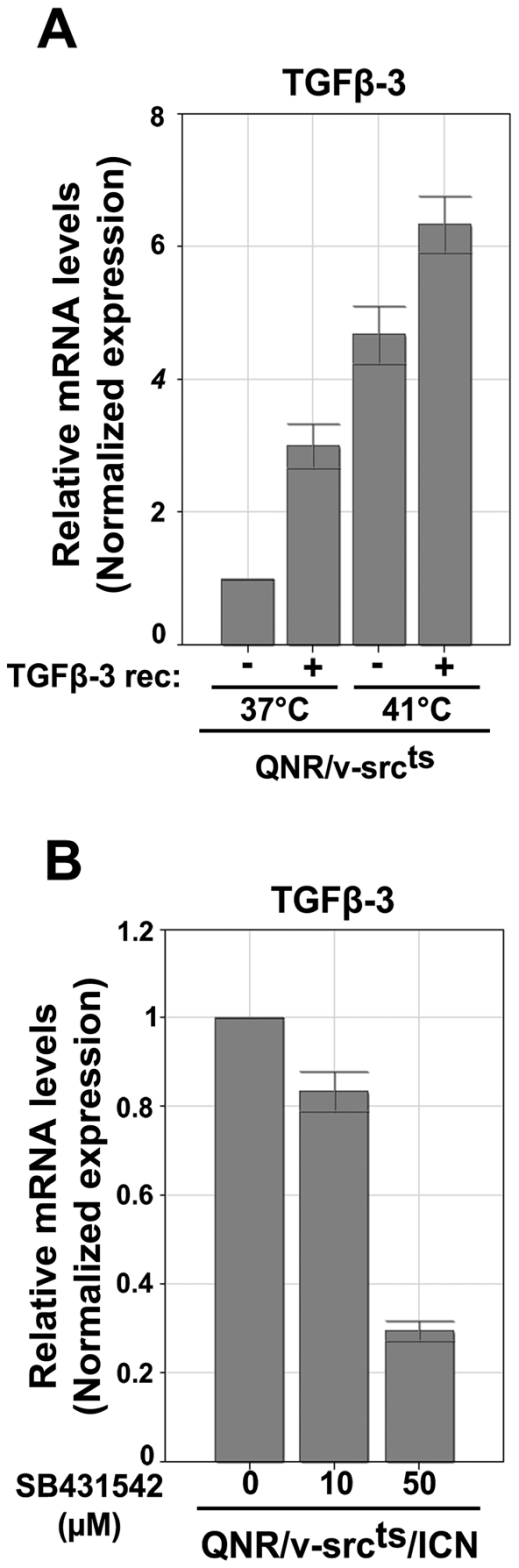
TGF-β3 expression is controlled by a positive feed-back loop. QPCR analysis of TGF-β-3 mRNA levels in (A) QNR/v-src^ts^ treated with 2 µg/ml of recombinant TGF-β3 or (B) QNR/v-Src^ts^/ICN cells at 37°C incubated with increasing doses of SB431542 during 24 hrs. After reverse transcription of 1 µg of RNA, TGF-β3 cDNA were amplified by QPCR. Results were normalized based on HPRT and TBP transcript levels and presented in relative arbitrary units using values obtained for untreated cells as reference equal to 1. Each bar represents the mean −/+ S.E. of three experiments.

TGF-β signaling was previously shown to directly regulate TGF-β3 transcription [19]. Therefore, we examined whether activation of this pathway in QNR/v-Src^ts^/ICN cells could be associated with an auto-regulation of TGF-β3 expression. We found that treatment of QNR/v-Src^ts^ cells with recombinant TGF-β3 induced an increase of TGF-β3 mRNA levels (Fig. 3A). Conversely, inhibition of TGF-β signaling in QNR/v-Src^ts^/ICN cells resulted in a decrease of TGF-β3 mRNA amounts (Fig. 3B). Therefore, it appears that TGF-β3 mRNA induction results in both mature TGF-β3 secretion and autocrine signaling activation, which in turn initiates a positive feed-back control maintaining the levels of TGF-β3 mRNA in QNR/v-src^ts^/ICN cells.

### TGF-β signaling suppresses v-Src-induced morphological transformation by restoring cytoskeleton organization

**Figure 4:**
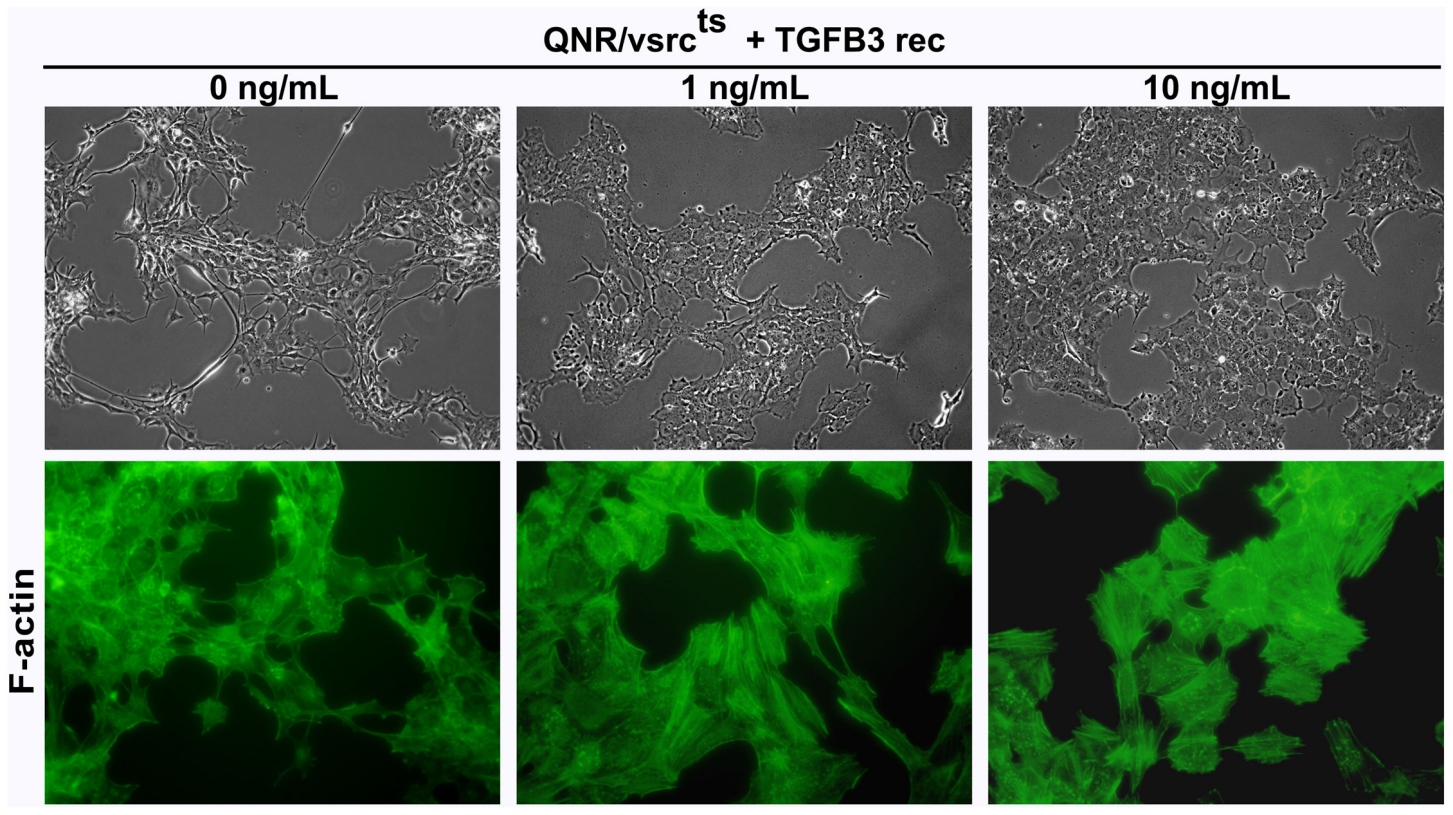
Recombinant TGF-β3 suppresses morphological transformation and induces cytoskeleton reorganization. QNR/v-src^ts^ cells were incubated at 37°C in absence or presence of 1 or 10 ng/ml of recombinant TGF-β3 protein. 24 hrs later, cells were fixed and F-actin stress fibers labeled with FITC-phalloïdin. Magnification: x20 for phase-contrast morphology (a–c); x40 for F-actin staining (d–f).

To determine whether suppression of morphological transformation was due to TGF-β3 activity, we examined the effects of recombinant TGF-β3 on cell morphology. QNR/v-src^ts^ cultures, maintained at 37°C, are typically composed of refractile round or fusiform transformed cells. Their morphology was markedly changed within 24 hours, following treatment with 1–10 ng/ml of recombinant TGF-β3 (Fig. 4 a–c). Cells became flat, transparent with a polygonal, epithelial like morphology. The extent of these changes reflected a major cytoskeleton reorganization. Transformation of QNR/v-src^ts^ cells is defined by the loss of actin stress fibers (Fig. 4 d). In contrast, the cytoskeleton of TGF-β3 treated cells became well organized, with abundant stress fibers (Fig. 4 e, f).

**Figure 5:**
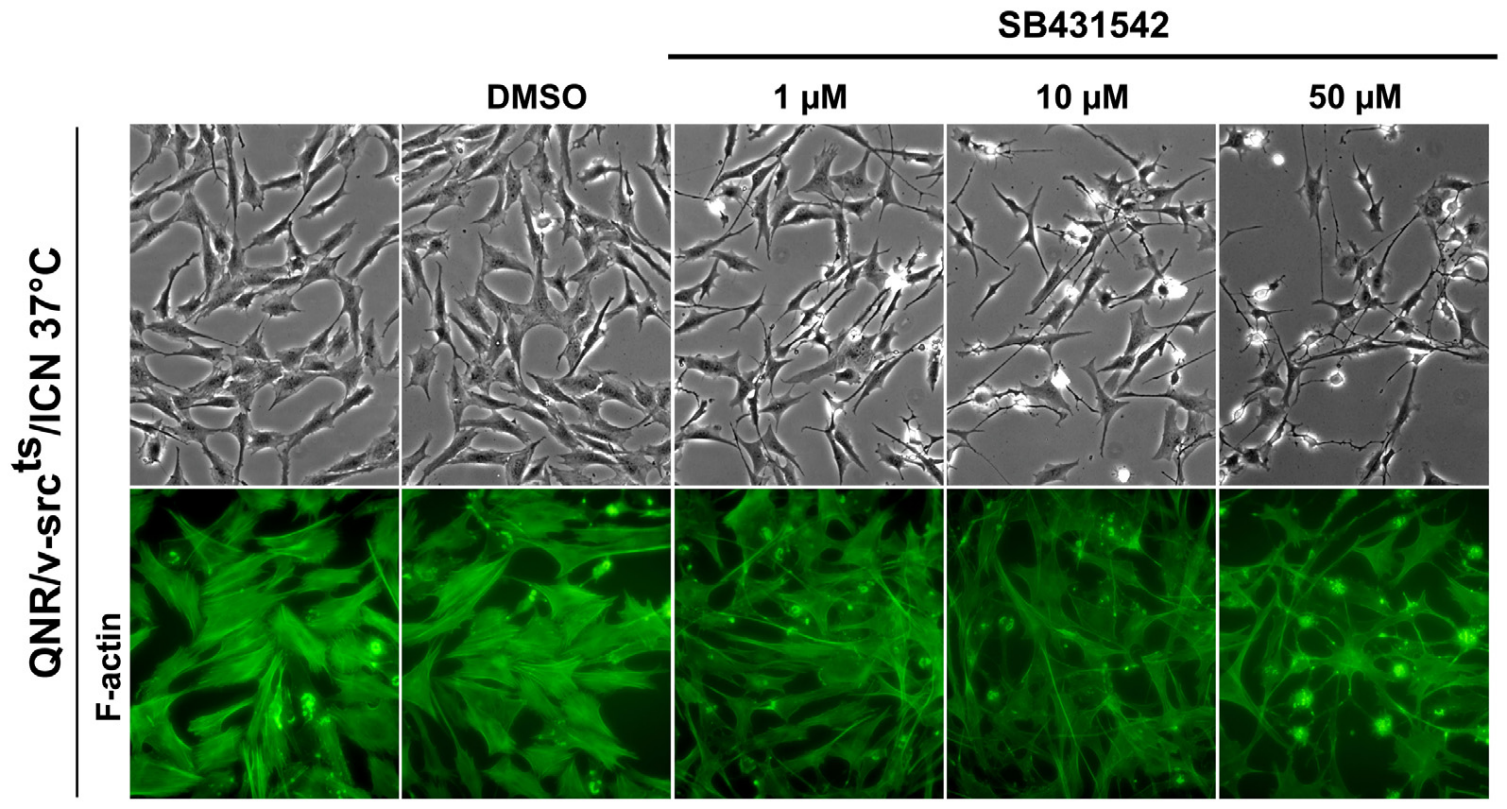
Inhibition of TGF-β signaling disrupts cell morphology and cytoskeleton organization of QNR/v-Src^ts^/ICN cells. QNR/v-Src^ts^/ICN cells were incubated at 37°C in presence of DMSO (control) or increasing (1–50 µM) concentrations of SB4314542. SB431542 treatment induces retraction of cells but no toxicity even at high doses. 3 days later, cells were fixed and F-actin stress fibers were labeled with FITC-phalloïdin. Magnification: x20 for morphology (a–e); x40 for F-actin staining (f–j).

QNR/v-src^ts^/ICN cells maintained at 41°C exhibit a normal morphology. When transferred to 37°C, they retain an organized cytoskeleton underlined by the presence of numerous stress fibers [2]. We investigated whether autocrine TGF-β signaling was responsible for maintaining their normal morphology, in presence of an active oncoprotein. Treatment of these cells with increasing concentrations of SB431542 induced morphological changes, including a marked increase in the number of refractile cells extending processes (Fig. 5 a–e). Actin fibers became thinner and less organized in treated cells (Fig. 5 h–j). These changes were not detected in control cells treated with DMSO (Fig. 5 f, g). Similar treatment of QNR/v-src^ts^ cells had no detectable effect on their morphology (data not shown). These results indicated a dominant interference of TGF-β signaling with cytoskeleton organization, to restore a normal cell morphology.

### TGF-β signaling suppresses morphological transformation by increasing α2-actin expression and Myosin Light Chain phosphorylation

**Figure 6:**
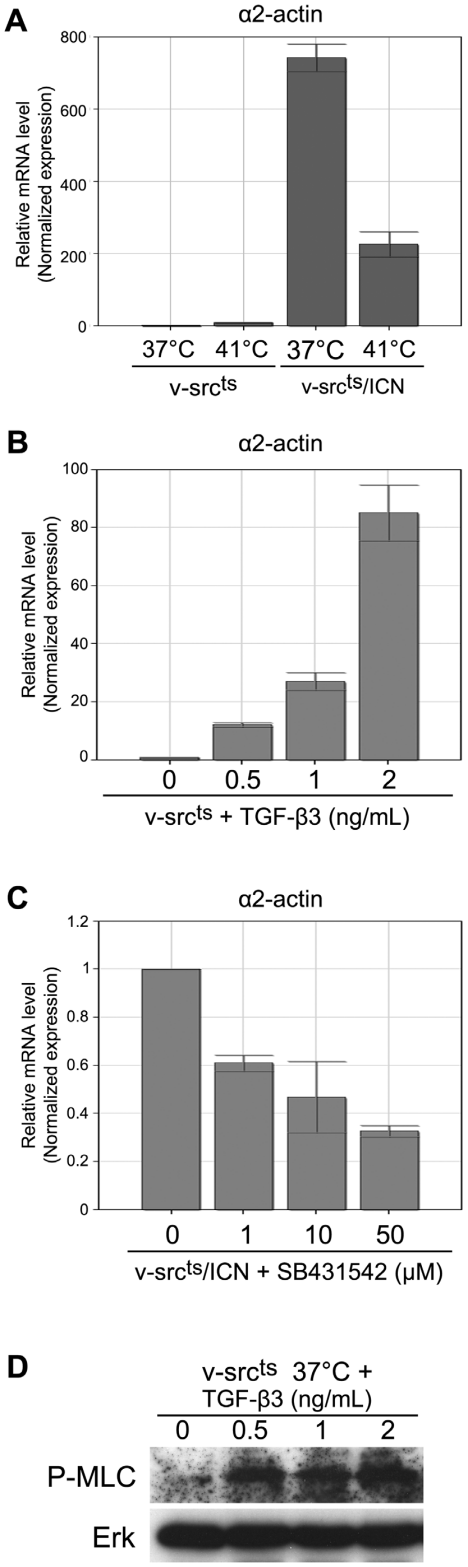
TGF-β signaling induces α2-actin expression and MLC2 phosphorylation. QPCR analysis of α2-actin mRNA levels in (A) QNR/v-src^ts^ and QNR/v-src^ts^/ICN cells at 37°C and 41°C, (B) QNR/v-src^ts^ cells treated at 37°C for 24 hrs with increasing concentrations (0.5–2 ng/ml) of recombinant TGF-β3 protein, or (C) QNR/v-src^ts^/ICN treated at 37°C for 24 hrs with increasing concentrations of SB431452 (1–50 µM) Results are represented in relative arbitrary units after normalization using HPRT and TBP transcript levels. For (A), data presented are representative of three distinct experiments. For (B and C), each bar represents the mean −/+ S.E. of three experiments. (D) QNR/v-src^ts^ cells were treated with increasing doses of recombinant TGF-β3 protein for 24 hrs. One hour before lysis, TGF-β3 treatment was renewed. Phosphorylated MLC2 was detected by western-blot and Erk levels were used for normalization.

TGF-β activity was previously shown to interact with signaling pathways targeting cytoskeleton components (for review [20]). Accordingly, our comparative RNA analysis showed a 25-fold increase of α2-actin mRNA in QNR/v-src^ts^ cells stably expressing ICN, as compared to v-Src-transformed cells. This was validated by QPCR (Fig. 6A), suggesting that TGF-β signaling contributes to cytoskeleton restoration, in part by increasing the abundance of α-actin fibers. Moreover, treatment of QNR/v-src^ts^ cells with recombinant TGF-β3 significantly increased the levels of α2-actin mRNA, as determined by QPCR (Fig. 6B). Conversely, treatment of QNR/v-src^ts^/ICN cells with SB431542 resulted in a decreased α2-actin expression (Fig. 6C), indicating a significant contribution of TGF-β signaling in regulating the levels of this mRNA in ICN containing cells.

We previously correlated suppression of morphological transformation, in QNR/v-src^ts^/ICN cells, with an increase of Myosin Light Chain (MLC) phosphorylation, as compared to v-Src transformed cells [2]. To assess the contribution of TGF-β signaling to this phosphorylation, we treated QNR/v-src^ts^ cells with increasing doses of recombinant TGF-β3. This treatment increased the levels of MLC phosphorylation in QNR/v-src^ts^ cells, as compared to untreated cells (Fig. 6D).

Taken together, these results indicate that TGF-β signaling contributes to cytoskeleton reorganization both by increasing expression of α2-actin and favoring its polymerization into stress fibers by inducing MLC phosphorylation [21].

### Autocrine TGF-β signaling contributes to anchorage-independent growth inhibition of QNR/v-src^ts^/ICN cells

**Figure 7:**
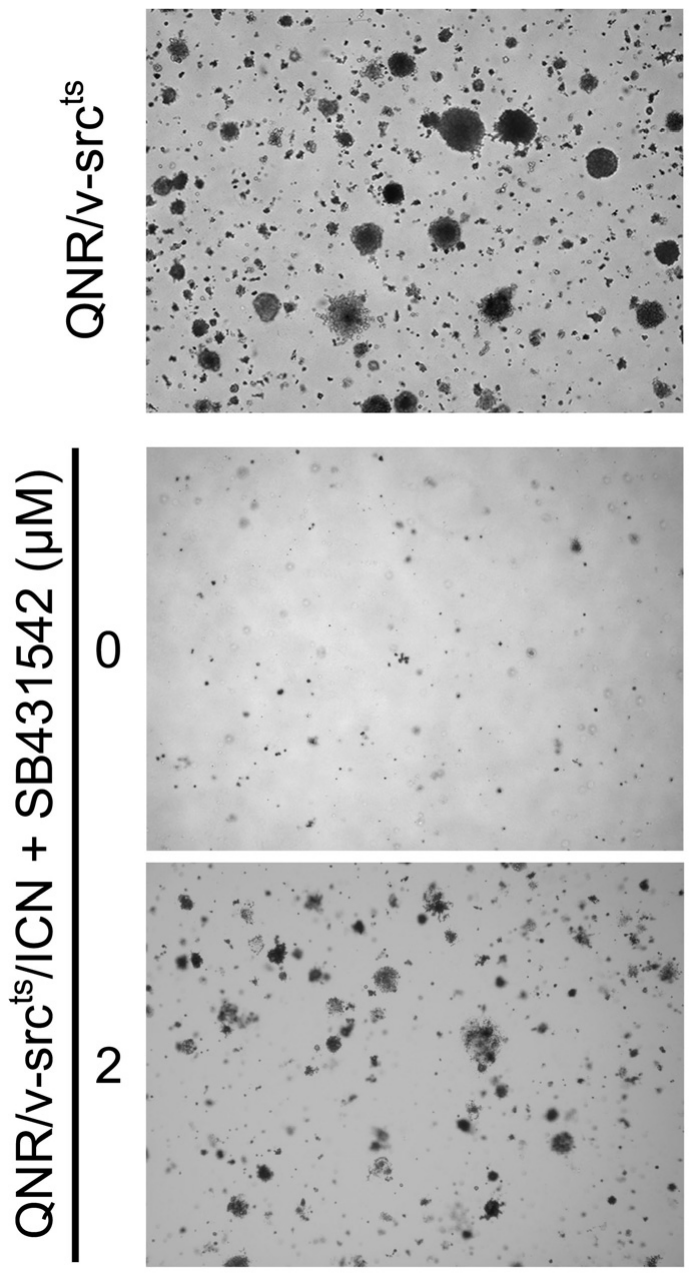
Inhibition of TGF-β signaling restores anchorage-independent growth of QNR/v-Src^ts^/ICN cells. QNR/v-Src^ts^/ICN cells were incubated without or with 2 µM of SB431542 for 24 hrs and subsequently seeded in soft agar containing medium, in absence or presence of SB431542. Treatment with the inhibitor was renewed every second day during 2 weeks. Pictures of colonies were taken 15 days later. Magnification x20.

In addition to morphological transformation, v-Src transformed QNR cells display anchorage-independent growth capacity [8], a characteristic marker of *ex-vivo* oncogenic transformation. QNR/v-src^ts^ cells efficiently give rise to colonies in soft agar containing medium, whereas QNR/v-src^ts^/ICN cells fail to divide under the same conditions. Moreover, treatment of QNR/v-src^ts^ cells with ICN medium also inhibits their anchorage-independent growth capacity [2]. We investigated whether autocrine TGF-β activity was also responsible for the loss of anchorage-independent growth by QNR/v-src^ts^/ICN cells. Therefore, we tested their capacity to form colonies in soft agar containing medium, in presence or absence of the SB431542 inhibitor. We found that while untreated cells could not give rise to colonies, their anchorage-independent growth capacity was restored when TGF-β signaling was inhibited. However, this restoration was only partial, as the number and size of colonies were smaller than those observed in QNR/v-src^ts^ cells (Fig. 7).

Therefore, activation of autocrine TGF-β signaling, following ICN stable expression, is predominantly responsible for suppressing v-Src-induced transformation of QNR cells, as defined by the loss of both morphological transformation and anchorage-independent growth capacity.

### TGF-β3 signaling is required to induce and maintain expression of glial markers in QNR/v-src^ts^/ICN cells

We previously showed that primary dissociated QNR cultures are composed of a mixture of progenitor and differentiated cells. Accordingly, they contain neuronal and glial markers, indicating that they can autonomously proceed into differentiation (32). As a result of transformation, this process is interrupted, as the vast majority of QNR/v-src^ts^ cells express only the marker combination of progenitor cells. In QNR/v-src^ts^/ICN cells, the loss of precursor cell markers was combined with the acquisition of neuroretina glial cell markers, indicating a switch in differentiation program (32).

**Figure 8:**
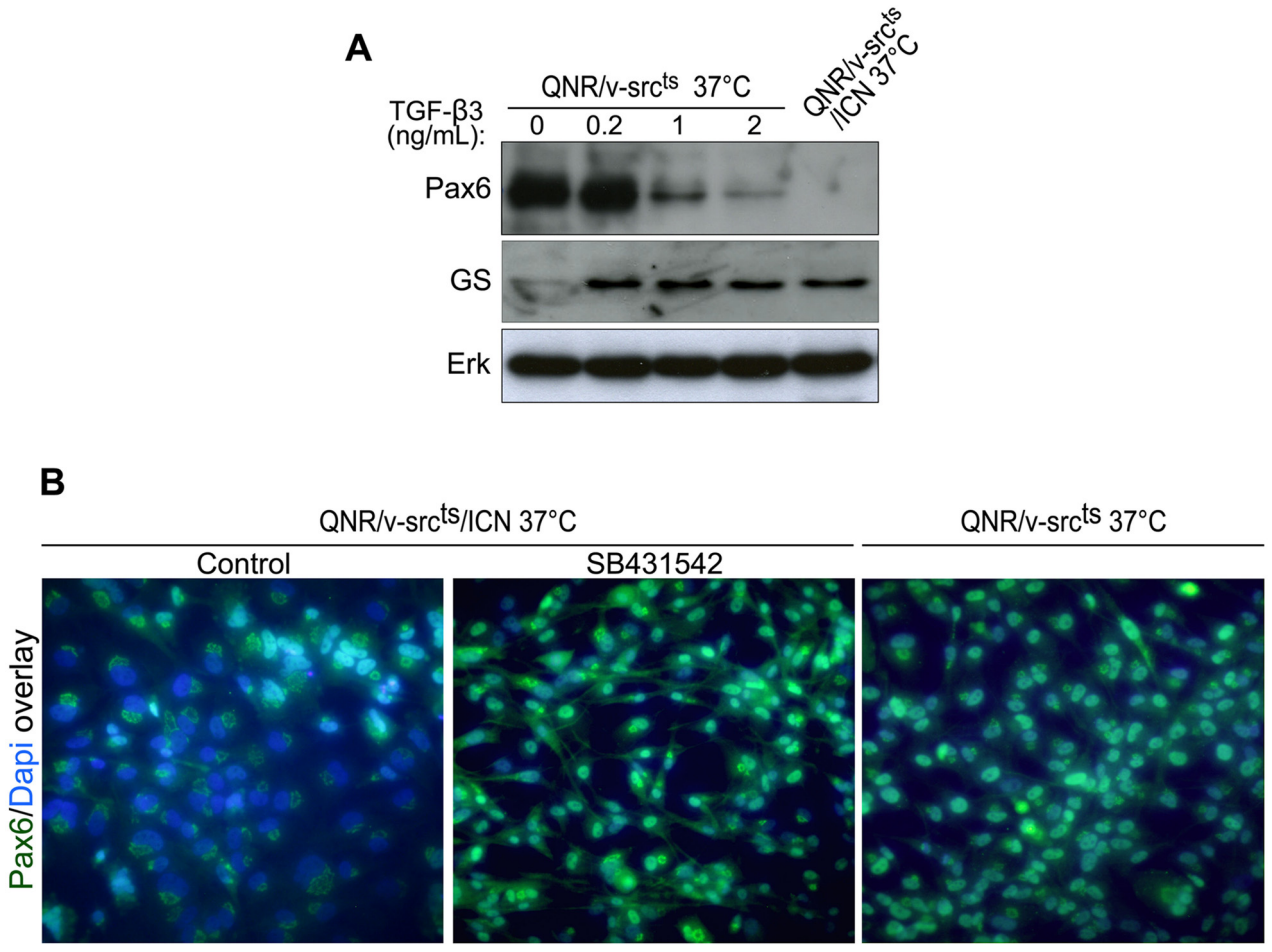
TGF-β signaling controls commitment into glial differentiation. (A) QNR/v-src^ts^ cells were treated at 37°C with increasing concentrations (0.2–2 ng/ml) of recombinant TGF-β 3 protein during 7 days. Pax6 and Glutamine Synthetase (GS) were detected by western-blot. Protein loading was normalized using Erk antiserum. (B) Pax6 expression was analyzed by immunofluorescence after treatment of QNR/v-Src^ts^/ICN cells at 37°C with DMSO (control) or 10 µM SB431542 during 7 days. The majority of control QNR/v-Src^ts^/ICN cells did not express nuclear Pax6 but we observed a weak peri-nuclear labeling. Magnification x40.

To determine the contribution of TGF-β signaling to these changes, we treated QNR/v-src^ts^ cells with recombinant TGF-β3 and examined the relative expression of two markers: Pax6, predominantly present in progenitor and transformed cells [2] and glutamine synthetase (GS), a marker of mature glial cells [22]. We observed a marked decrease of Pax6 protein levels together with an induction of GS expression, in treated cells (Fig. 8A). Thus, activation of TGF-β signaling is sufficient to inverse the balance of differentiation markers in QNR/v-src^ts^ cells and recapitulates the opposite evolution of Pax6 and GS markers, as observed both during QNR cell differentiation and in QNR/v-src^ts^/ICN cells [22], [23].

We next examined whether autocrine TGF-β signaling was responsible for inducing and maintaining glial like differentiation of QNR/v-src^ts^/ICN cells. Therefore, we treated these cells with SB431542, once ICN stable expression was achieved, and examined whether they could be induced to reexpress Pax6. We found that while the number of nuclear Pax6 positive cells was initially low in these cultures, the proportion of cells containing this marker markedly increased, following TGF-β signaling inhibition (Fig. 8B). Conversely, GS expression was suppressed in treated cells (data not shown). These results indicate that autocrine activation of TGF-β signaling, as a consequence of ICN stable expression, is effective in upsetting the balance of markers expression in QNR/v-Src^ts^ cells.

### TGF-β3 mRNA levels increase during ex-vivo QNR cell differentiation

**Figure 9:**
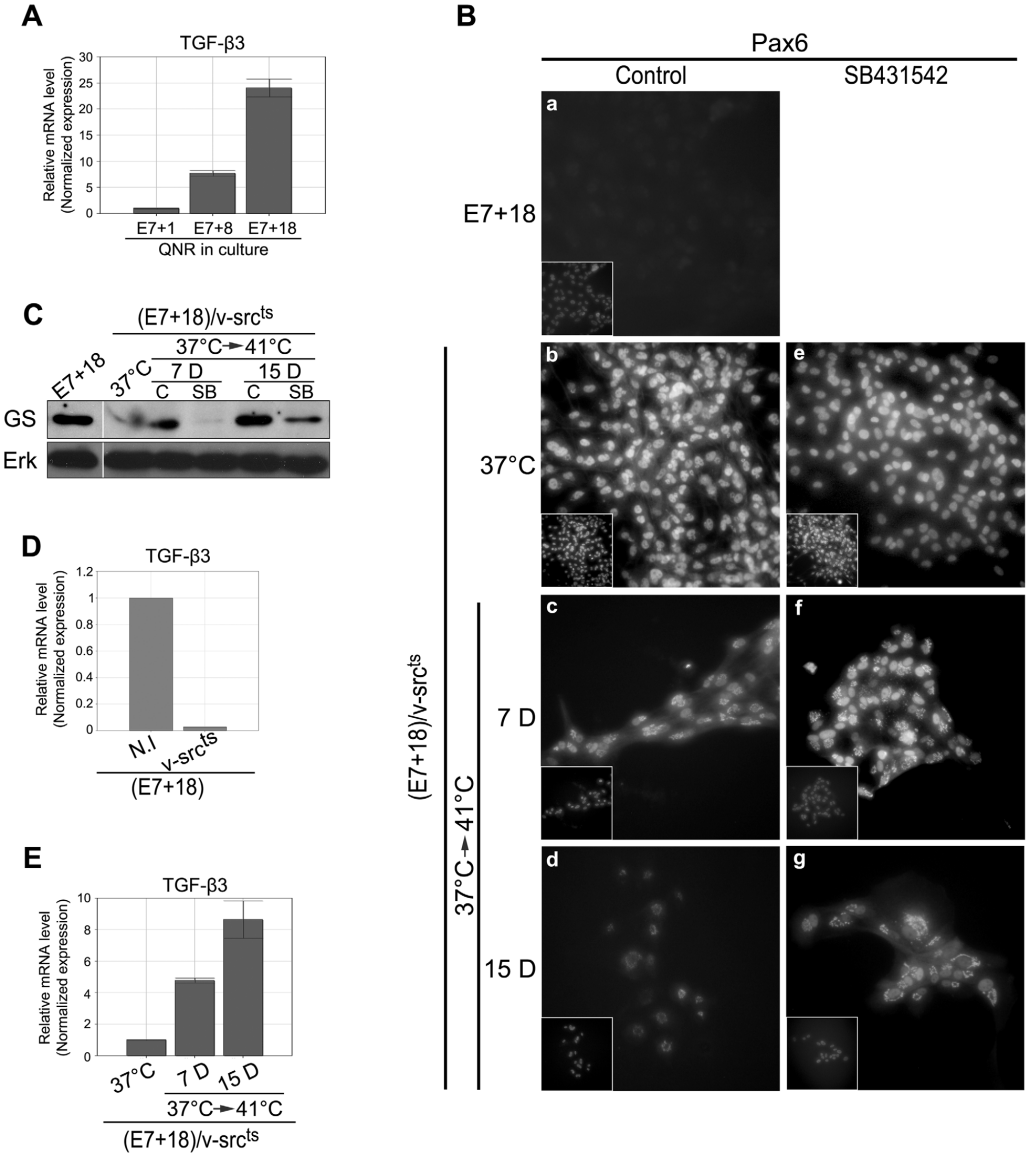
Inhibition of QNR cell differentiation correlates with TGF-β3 down-regulation by v-Src. (A) QPCR analysis of TGF-β3 mRNA levels in 7-day-old (E7) embryonic QNR seeded for 1 (E7+1), 8 (E7+8) or 18 (E7+18) days. Results were normalized based on HPRT and TBP transcript levels and presented in relative arbitrary units, using values obtained for E7+1 QNR cells as reference. (B) Detection of Pax6 expression by immunofluorescence in QNR (E7+18) (a) and QNR(E7+18)/v-src^ts^ cells, maintained at 37°C (b-e) or transferred to 41°C for 7 (c–f) and 15 days (d–g), in presence of DMSO (control) or 10 µM SB431542. At 41°C (c–d), nuclear Pax6 expression progressively decreased in control cells, but we reproducibly observed an unusual Pax6 peri-nuclear labeling. Insets represent DAPI staining. Magnification x40 (C) Expression of Glutamine synthetase (GS) in QNR (E7+18) and QNR (E7+18)/v-src^ts^ cells at 37°C or following their transfer to 41°C for 7 and 15 days, in presence of DMSO (c) or 10 µM of SB431542 (SB) Erk was used to normalize protein loading. (D) TGF-β3 mRNA levels were analyzed by QPCR in uninfected (N.I) QNR (E7+18) cells or in QNR (E7+18)/v-src^ts^ cells at 37°C. (E) TGF-β3 mRNA levels in QNR(E7+18)/v-src^ts^ cells at 37°C or transferred to 41°C for 7 and 15 days. Values are represented in relative arbitrary units after HPRT and TBP normalization.

We then examined whether TGF-β3 activation in QNR/v-src^ts^/ICN cells was merely due to ectopic ICN expression or whether it belonged to restoration of the QNR *ex-vivo* differentiation program, as a consequence of Notch signaling. Therefore, we investigated the presence of TGF-β3 mRNA in QNR cells dissected from 7 day-old embryos (E7) and cultured for various time lengths. As mentioned above, these cells undergo an intrinsic differentiation program defined by the progressive loss of precursor cell markers and the acquisition of glial and neuronal cell markers [2]–[4]. We found that, while TGF-β3 mRNA was barely detectable in undifferentiated precursor cells one day after dissection (E7+1), its level progressively increased between 8 (E7+8) and 18 (E7+18) days, thereafter (Fig. 9A). These (E7+18) long term cultures essentially contain cells expressing only markers of glial differentiation (Fig. 9C). This indicated that steady state levels of TGF-β3 mRNA autonomously increase in cultured QNR cells and that this increasing parallels the course of their ex-vivo differentiation.

### v-Src-mediated transformation correlates with inhibition of ex-vivo differentiation and TGF-β3 down-regulation

Results described above indicated that TGF-β3 expression was down-regulated in both QNR progenitors and undifferentiated transformed QNR/v-Src^ts^ cells. The latter may simply reflect the possibility that precursor cells constitute a preferential target for v-Src-induced transformation and are subsequently maintained in their undifferentiated state, as a consequence of their sustained division. To determine whether v-Src activity would affect differentiation markers and TGF-β3 expression once established, we infected (E7+18) QNR cells, with RSVts68 and selected for transformed cells by passaging. While Pax6, a marker of precursor QNR cells, was no longer detected in (E7+18) uninfected cells (Fig. 9B a), the great majority of transformed cells reexpressed Pax6 (Fig. 9B b). In turn, the number of Pax6 positive cells progressively declined between 7 and 15 days, following v-Src thermal inactivation (Fig. 9B c,d), as observed during intrinsic ex-vivo differentiation (data not shown). Conversely, the GS glial marker, present in (E7+18) QNR cells, was no longer found in transformed cells, but became again detectable following v-Src inactivation (Fig. 9C).

We also found that the level of TGF-β3 mRNA was markedly reduced in transformed cells, as compared to that of uninfected cells (Fig. 9D), and progressively resumed following v-Src inactivation at 41°C (Fig. 9E). To determine the contribution of TGF-β signaling in regulating expression of these two markers, we treated (E7+18) QNR transformed cells with SB431542, prior to their transfer to the non-permissive temperature and maintained TGF-β signaling inhibition during 15 days. This resulted in delaying the autonomous down-regulation of Pax6 (Fig. 9B, e–g) and reexpression of GS (Fig. 9C).

Taken together, these results indicate that v-Src-mediated transformation of QNR cells, already displaying characteristics of glial cells, leads to repression of the GS marker and, interestingly, to the reemergence of undifferentiated cells expressing the Pax6 precursor marker. Conversely, oncoprotein inactivation allows these cells to resume their autonomous differentiation. Moreover, concomitant down-regulation of TGF-β3 expression appears to be strictly correlated with transformation of QNR cells and partially mediate the differentiation block. Therefore, concomitant arrest of differentiation and down-regulation of TGF-β3 appear essential to achieve transformation of QNR/v-src^ts^ cells. Finally, they suggest that TGF-β3 expression, which is restored in QNR/v-src^ts^/ICN cells, is constitutive of and effects their switch toward glial like cells.

## Discussion

We have been investigating how blocking differentiation contributes to the oncogenic transformation of neural cells and the possibility to activate instructive signals, as a mean to reverse the oncogenic process. In this work, we reported that v-Src-mediated transformation of QNR cells is strictly correlated with interruption of differentiation and maintenance of transformed cells in an undifferentiated state. In addition, transformation can be suppressed by restoring endogenous signaling pathways involved in determining cell identity. We identified autocrine activation of TGF-β3 signaling as a key effector of ICN-mediated switch in expression of differentiation markers and suppression of transformation.

*Ex-vivo* differentiating QNR cells undergo phenotypic changes that reflect the execution of an intrinsic program, characterized by the progressive loss of precursor cell markers (e.g. Pax6) and the acquisition of neuronal and glial differentiation markers [2]–[4]. Long term cultures become essentially composed of flat epithelial like cells, expressing only markers of glial differentiation (e.g. GS and vimentin). We showed that a marked increase in TGF-β3 mRNA steady state levels is part of this autonomous process and partially contributes to its execution. However, these quiescent QNR cultures display remarkable plasticity, as they can be transformed by v-Src, reenter the cell cycle and reexpress Pax6. Upon v-Src^ts^ thermal inactivation, their capacity to differentiate is again restored. Switching up and down the ratio of Pax6 to GS markers, depending on v-Src activity, correlates with down or up-regulation of TGF-β3 expression. Thus, in this cell system, concomitant down-regulation of differentiation markers and TGF-β3 expression proved essential for v-Src-induced transformation. Similar down-regulation of TGF-β3 transcription was reported in v-Src transformed chicken fibroblasts, without further assessment of its contribution to transformation [24]. The mechanisms leading to TGF-β3 down-regulation, as a consequence of v-Src activity, are presently unknown. This contrasts with other cell systems, where v-Src was shown instead to activate TGF-β1 transcription essentially through AP1 sites on its promoter [25] and with reports showing increased TGF-β activity in oncogene transformed cells [26], [27].

In contrast with v-Src-transformed cells, TGF-β3 mRNA is up-regulated in QNR/v-src^ts^/ICN cells, once ICN stable expression is established, and is maintained through multiple cell passaging despite the presence of an active oncoprotein. Our results indicate that TGF-β3 expression is regulated at multiple levels. First, TGF-β3 mRNA level in QNR/v-src^ts^/ICN cells remains partially under v-Src control, as it is significantly higher when the oncoprotein is inactive. This suggests the existence of a dominant negative interference of Notch signaling with v-Src-dependent TGF-β3 down-regulation. Second, increased TGF-β3 expression also appears to be a consequence of Notch signaling *per se*, as its mRNA is maintained at a significantly higher level in QNR/v-src^ts^/ICN as compared to QNR/v-src^ts^ cells, when v-Src^ts^ is thermally inactivated and Notch signaling is acting alone. However, we found that TGF-β3 mRNA levels were not significantly diminished when ICN expression was down-regulated by specific siRNA, in QNR/v-Src^ts^/ICN cells. Similarly, forced ICN expression in QNR/v-Src^ts^ cells did not increase these levels in transient transfection assays (data not shown). Therefore, TGF-β3 mRNA up-regulation in QNR/v-Src^ts^/ICN cells is not a direct consequence of ICN expression but is likely to belong to the restoration of their autonomous differentiation program, as observed in normal QNR cells, and is subsequently maintained through a positive feed-back control. Although the mechanism involved in TGF-β3 mRNA up-regulation remains undefined, our work provides the first example of Notch and TGF-β signaling cooperation in suppressing transformation.

Mature TGF-β3 is secreted by QNR/v-src^ts^/ICN cells and initiates an autocrine loop to activate endogenous TGF-β signaling. Analyzing how this signaling contributes to the phenotypic changes induced by ICN, we have established a strong correlation between its capacity to suppress transformation and induce the switch in differentiation markers. Thus, treatment of precursor like QNR/v-src^ts^ cells with recombinant TGF-β3 is sufficient to convert them into glial like cells, by down-regulating expression of Pax6 and inducing that of GS. This is concomitant with the loss of their transformed phenotype. Our data also point to a major role of TGF-β signaling in the control of cell morphology and cytoskeleton organization, as also reported in other cell systems (for review [20]). Maintaining normal cell morphology may also be essential for differentiation. This is supported by previous data showing that cytoskeleton depolymerization inhibits expression of the GS marker of Müller cell differentiation [28]. Thus, TGF-β signaling would control differentiation by acting both on cell markers expression and cytoskeleton organization. Therefore, it appears that the majority of the phenotypic changes, induced by ICN stable expression, are achieved by restoring endogenous TGF-β signaling, repressed as a consequence of v-Src activity.

Both Notch and TGF-β signaling play a dual role in oncogenesis, depending on the cell context (for review [14], [29]. Notch activation contributes to oncogenesis by promoting survival or proliferation of undifferentiated precursor cells. The role of TGF-β signaling is more complex and impacts on various steps of tumor progression (for review [16], [30]. When acting as tumor suppressors, both pathways promote growth arrest by upregulating expression of the cyclin/CDK inhibitor p21^cip1/waf1^, which in turn favors differentiation [31], [32]. Our results indicate that suppression of QNR/v-src^ts^ cell transformation by the conjunction of both signaling pathways involves distinct mechanisms, in various aspects. First, in this cell system, QNR/v-src^ts^ cells reversion to a normal phenotype, in presence of an active v-Src, is compatible with their sustained division. Moreover, ICN stable expression confers extended growth capacity upon QNR/v-src^ts^/ICN cells when v-Src^ts^ is thermally inactivated, whereas QNR/v-src^ts^ cells stop dividing under these non permissive conditions (32). This strongly suggests that, while QNR/v-src^ts^/ICN cells have acquired glial specific markers, they are not mature glial cells, as their terminal differentiation would normally require extinction of Notch signaling [33], [34]. Second, we also reported a strong cytoplasmic accumulation of p21^cip1/waf1^ in dividing QNR/v-src^ts^/ICN cells, suggesting for this protein a role distinct from that of a cell cycle inhibitor [2]. Third, all these phenotypic changes take place in presence of an active oncoprotein, in contrast to other models where oncogene inactivation is required for differentiation [35]–[37].

Tumor cells generally do not respond to extracellular signals that normally control their growth or differentiation [38]. A likely reason would be that they down-regulate expression of genes encoding such secreted proteins or their receptors. Alternatively, proteins secreted by tumor cells could be endowed with pleiotropic properties and instead favor tumor progression, as described with TGF-β signaling (for review [16], [39]. However, it was also shown that certain tumor cells display remarkable plasticity when placed in appropriate environments (for review, [40], [41]. Therefore, analyzing the response of tumor cells to extracellular signals, potentially capable of modulating the transformed phenotype is required to understand crucial events in transformation and develop specific therapeutic approaches.

## Materials and Methods

### Reagents

The TGF-β type I receptor kinase inhibitor, SB431542 was purchased from Sigma (Sigma-Aldrich, Lyon, France) and reconstituted in dimethylsulfoxide (DMSO). TGF-β3 recombinant protein (243-B3) and TGF-β3 monoclonal antibody (MAB243) were purchased from R&D systems (Minneapolis, MN, USA).

### Cell cultures and virus

QNR were dissected from 7-day-old Japanese quail embryos (Cailles de Chanteloup, Corps-Nuds, France) and cultured in Basal Medium Eagle (BME) supplemented with 8% fetal calf serum (Biowest, Nuaillé, France), as described (Pessac et al., 1983). QNR cells were infected with Rous Sarcoma Virus tsNY68 encoding a thermolabile mutant of v-Src. Isolation and properties of RSV tsNY68 mutant were reported previously (Kawai and Hanafusa, 1971). QNR/v-src^ts^/ICN were obtained as previously described [2].

### Preparation of cell-conditioned media

QNR/v-src^ts^ and QNR/v-src^ts^/ICN cells (2.10^6^ cells/100 mm dish) were cultured in serum-free BME. Serum-free media were collected after 30 hours and filtered. When necessary, collected supernatants were supplemented with 8% FCS.

### Anchorage-independent growth

Colony formation in soft agar was assessed as previously described [2]. For paracrine activity assays, cell-free culture supernatants were diluted 1∶2 with BME and supplemented with DMSO or 2 µM of SB431542. Every second day, media were renewed in presence of DMSO or 2 µM of SB431542. Colonies were observed 10–15 days later.

### RNA preparation and Microarray RT-PCR analysis

Total RNA was prepared using the RNeasy extraction kit (Qiagen, Courtaboeuf, France). 1 µg of total RNA was reverse-transcribed into cDNA using Superscript II reverse transcriptase (Invitrogen SARL, Cergy-Pontoise, France). Quantitative real-time PCR (QPCR) analyses were performed with iQ SYBR Green supermix (Bio-Rad, Marnes-la-coquette, France). Primers used for real-time QPCR were designed according to sequences in GenBank (Table 1). Reactions were carried out in an iCycler Thermal Cycler (Bio-Rad, Marnes-la-coquette, France) for 40 cycles (95°C for 15 sec, 60°C for 1 min) after an initial 10-min incubation at 95°C. mRNA levels were normalized to the levels of HPRT and TBP mRNA.

**Table 1:** Sequence of primers used to amplify specific mRNA by QPCR.

Gene	Sense Primers	Antisense Primers	Acess number
TGF-β3	5′-GGAGGAGGAGAAGGAGGAGA-3′	5′-CAAATGCCCAACTCATTGTG-3′	NM_205454
α2-actin	5′-CACCCAACTCTGCTGACTGA-3′	5′-ATACATGGCTGGCACATTGA-3′	NM_001031229
HPRT	5′-CGCCCTCGACTACAATGAAT-3′	5′-TACTTCTGCTTCCCCGTCTC-3′	NM_204848
TBP	5′-AAGCGACACAGGGAACATCT-3′	5′-GACGGGTACAGAGGTGTGGT-3′	NM_205103

Each set of forward and reverse primers was designed from NCBI published sequences using Primer3 website (http://frodo.wi.mit.edu/cgi-bin/primer3/primer3_www.cgi).

For microarray analysis, total RNA was analyzed and processed by the genomic facility of the Fred Hutchinson Cancer Research Center (Seattle, WA, USA) using a 13,000 chicken microarray (http://www.fhcrc.org/science/shared_resources/genomics/dna_array). Genes were considered to be differentially regulated when the log2 ratio between two conditions and in the dye swap experiments was superior to 0.58 corresponding to a variation of 1.5. All the gene array data has been deposited in the GEO database (GSE22054).

### Western blotting

Cell extracts preparation, protein analysis by SDS-PAGE and western-blotting were performed as previously reported [2]. Blots were probed with primary antibodies used at the following dilutions: anti-phosphorylated Smad2 (Ser465/467; #3101) and anti-phosphorylated MLC2 (Ser19; #367) polyclonal antibodies (Cell Signaling Technology, Hertfordshire, UK) at 1/1000^e^; anti-TGF-β3 monoclonal antibody (MAB243; R&D systems, Minneapolis, MN, USA) at 1/500^e^; anti-Glutamine Synthetase monoclonal antibody (Chemicon, Hampshire, UK) at 1/1000^e^; anti-Erk1 polyclonal antibody (SC-93, Santa-Cruz Biotechnology, Santa-Cruz, CA, USA) at 1/2000^e^; and anti β-actin monoclonal antibody (Amersham Pharmacia Biotech, Piscataway, NJ, USA) at 1/10000^e^.

### Immunofluorescence

Cells were fixed and treated as previously described [2]. Mouse monoclonal anti-Pax6 antibody (kindly provided by S Saule) was used at 1/10^e^. Fluorescein isothiocyanate (FITC)-conjugated goat anti-mouse immunoglobulin G (Sigma-Aldrich, Lyon, France) was diluted at 1/200^e^. F-actin fibers were labeled as described [2].
